# Supplementary material for: Tezacaftor/Ivacaftor therapy has negligible effects on the cystic fibrosis gut microbiome
Source: Microbiol Spectr. 2023 Aug 21;11(5):e01175-23. doi: 10.1128/spectrum.01175-23 (PMC10581179; doi:10.1128/spectrum.01175-23)
Supplement: Supplemental material — Supplemental methods and materials. [file spectrum.01175-23-s0001.pdf]

Supplementary Methods & Results

Tezacaftor/Ivacaftor therapy has negligible effects on the cystic fibrosis gut microbiome

Ryan Marsh [a], Claudio Dos Santos [b], Liam Hanson [b,c], Christabella Ng [d,e], Giles Major [d,f], Alan R Smyth [d,e], Damian Rivett [b #], Christopher van der Gast [a,g #]

[a] Department of Applied Sciences, Northumbria University, Newcastle, UK

[b] Department of Natural Sciences, Manchester Metropolitan University, Manchester, UK

[c] Department of Life Sciences, Manchester Metropolitan University, Manchester, UK

[d] School of Medicine, University of Nottingham, Nottingham, UK

[e] NIHR Nottingham Biomedical Research Centre, Nottingham, UK

[f] Nestlé Institute of Health Sciences, Société des Produits Nestlé, Lausanne, Switzerland

[g] Department of Respiratory Medicine, Salford Royal NHS Foundation Trust, Salford, UK

[#] Correspondence to: Professor Chris van der Gast, Department of Applied Sciences, Faculty of Health and Life Sciences, Ellison Building, Newcastle Upon Tyne, NE1 8ST, UK; chris.vandergast@northumbria.ac.uk. Dr Damian Rivett, Department of Natural Sciences, Faculty of Science and Engineering, John Dalton Building, Chester Street, Manchester, M1 5GD, UK; d.rivett@mmu.ac.uk

Supplementary Methods

Study participants and design

Fourteen pwCF, homozygous for p.Phe508del, were initially recruited from Nottingham University Hospitals NHS Trust. Participants were asked to provide stool samples whilst attending the Sir Peter Mansfield Imaging Centre at the University of Nottingham. Ultimately, 12 pwCF (mean age at baseline, 20.8 ± 7.80 years, median age, 20 years) provided samples available for inclusion in our analyses. Participants were asked to fast overnight and prior to the refraining from the use of laxatives and anti-diarrhoeals during their visit. Regular pancreatic enzyme replacement therapy and physiotherapy procedures were admitted.

Clinical assessments and sample collections were performed at baseline, with further samples collected following either Tezacaftor/Ivacaftor or an identical looking placebo administration, in a randomised double-blind two period-crossover fashion. Randomisation was carried out externally to those involved in analyses or in the management of patients in the study. Tezacaftor/Ivacaftor and placebo formulations were visually matched and coded to ensure blindness to investigators. Both treatments were administered for 28 days with an intermediate 28-day washout period. Tezacaftor/Ivacaftor was taken during the morning and Ivacaftor in the evening with patients advised to administer treatment alongside fat-containing foods. Between day 19-21 of each phase, participants provided faecal samples whilst at clinic, and completed the validated PAC-SYM and CF-Abd questionnaires to assess gut symptoms (1, 2).

Full crossover from baseline to both treatments (placebo and Tezacaftor/Ivacaftor) was achieved for 9/12 pwCF, with the remaining 3 patients receiving open label Tezacaftor/Ivacaftor treatment following baseline and 2 pwCF not completing the study in a capacity enabling subsequent microbiota analysis. Faecal samples from 10 age-matched healthy controls (mean age, 21.4 ± 7.40 years, median age, 20 years) from our previous study were available for microbiota and metabolomic comparison also (3). Further clinical trial details can be found at clinicaltrials.gov, under number NCT04006873 (https://clinicaltrials.gov/ct2/show/NCT04006873).

PMA treatment prior to DNA extraction

1 mg PMA (Biotium, CA, USA) was hydrated in 98 µL 20% dimethyl sulfoxide (DMSO) to give a working stock concentration of 20 mM. 300 mg of stool thawed out from -80 °C was homogenised in 3 ml PBS, and centrifuged at 3200 g, for 5 minutes. The pellet was then resuspended in 1 ml PBS prior to splitting into 500 µL fractions for subsequent PMA treatment. 1.25 µL of PMA (20 mM) was added to give a final concentration of 50 µM. Following the addition of PMA to samples in opaque Eppendorf tubes, PMA was mixed by vortexing for 10 seconds, followed by incubation for 15 minutes at room temperature (~ 20 °C). This step was repeated before the transfer of samples to clear 1.5 mL Eppendorf tubes and placement within a LED lightbox. Treatment occurred for 15 minutes to allow PMA intercalation into DNA from compromised bacterial cells. Samples were then centrifuged at 10,000 x g for 5 minutes. The supernatant was discarded, and the cellular pellet was resuspended in 200 µL PBS.

Targeted amplicon sequencing – Bacterial 16S rRNA

Step 1 amplicon generation with primers based on the universal primer sequences 515F & 926R as described by Walters et al. (4), was performed under the following conditions: Initial denaturation of 180 seconds at 98 ºC, followed by: 25 cycles of 30 seconds at 95 ºC, 30 seconds at 55 ºC and 30 seconds at 72 ºC. A final extension of 5 minutes at 72 ºC was also included to complete the reaction. Step 2, the addition of dual barcodes and Illumina adaptor sequences was performed under the following conditions: Initial denaturation of 30 seconds at 98 ºC, followed by: 10 cycles of 10 seconds at 98 ºC, 20 seconds at 62 ºC and 30 seconds at 72 ºC. A final extension of 2 minutes at 72 ºC was also included to complete this reaction. This resulted in the generation of an ~ 550 bp amplicon spanning the V4-V5 hypervariable regions of the 16S rRNA gene.

Sequencing Controls and Library Pooling

PCR & DNA extraction negative controls were implemented, alongside the use of mock community positive controls, which included a Gut Microbiome Standard (ZYMO RESEARCH™). Following Barcode attachment in the second PCR step, clean-up and subsequent amplicon size selection was performed with AMPure XP beads (Beckman Coulter™) and quantified using a Qubit™ dsDNA HS kit. Sample concentrations were then manually normalised, pooled, and diluted to the final library concentrations requited for use on the Illumina MiSeq system.

Sequence processing and analysis

DADA2 was used to demultiplex and remove primer sequences, validate the quality profiles of forward and reverse reads and subsequently trim, infer sequence variants, merge denoised paired-reads, remove chimeras, and finally assign taxonomy via Naive Bayesian Classifier implementation. This included the use of the Genome Taxonomy Database (GTDB) reference sequences (5). Unidentifiable ASVs were run through a BLAST (https://blast.ncbi.nlm.nih.gov/Blast.cgi) and matched appropriately based on query coverage where possible. Taxa with 2 ≥ reads for a single sample were removed and excluded from subsequent statistical analysis. ASVs from the same bacterial taxon were collapsed to form a single OTU for a given taxon.

GC-MS: Sample processing and SCFA preparation

Faecal samples stored at -80ºC were ground in liquid nitrogen before 50 mg of ground faeces was added to 500 µl MS-grade water. Samples were lysed and homogenised utilising inert ZR BashingBead Lysis Tubes (Cambridge bioscience, UK), using the FastPrep-24 5G instrument (MP Biomedicals, California, USA) with two cycles at a speed of 6.0 m/s for 40 seconds each. Samples were then incubated at 4 °C whilst mixing for 30 minutes using an ELMI Intelli-Mixer™ RM-2L at 80 rpm. Samples were centrifuged at 13,000 x g for 30 mins. The supernatant containing faecal SCFAs was removed. 150 µL of supernatant was protonated with 5M HCl before the addition of anhydrous diethyl ether (1:1 v/v), samples were vortexed for 10 seconds, and incubated on ice for 5 mins. Following incubation, samples were mixed with the ELMI Intelli-Mixer™ RM-2L as before for 15 minutes, then centrifuged at 10,000 x g for 5 mins. The DE layer containing faecal SCFAs was transferred to a new Eppendorf tube pre-loaded with 25 mg Na_2SO_4. The remaining layer was then re-extracted with another 150 µL DE as before. Samples were equally pooled and then 40 µl was then transferred to a GC-MS vial, with the addition of 2 µl N, O-bis(trimethylsilyl) trifluoroacetamide (BSTFA). Samples were vortexed then incubated for 3 hours at 37 °C before loading onto the GC-MS. MS grade water processed in parallel was used as a blank sample to correct the background.

GC-MS Analysis

GC-MS analysis was carried out using an Agilent 7890B/5977 Single Quadrupole Mass Selective Detector (MSD) (Agilent Technologies) equipped with a non-polar HP-5ms Ultra Inert capillary column (30 m × 0.25 mm × 0.25 µm) (Agilent Technologies). The Agilent 7693 Autosampler was used to inject 1.0 µL of the derivatised sample in triplicate at a split ratio of 20:1 at 265 °C, with a solvent delay of 2 minutes 30 seconds. The initial oven temperature was held at 40 °C for 2 minutes, followed by a 10 °C/min temperature ramp to 140 °C, then increased to 300 °C at the rate of 40 °C/min and kept at this temperature for 6 minutes. Electron impact (EI)

mode ionisation was utilised at 70 eV, with the instrumental parameters set at 230, 150 and 300 °C for source, quadrupole and interface temperatures, respectively. Selected ion monitoring (SIM) mode was used for quantification; all confirmation and target ions lists are summarised in Supplementary Table S6. Agilent MassHunter workstation version B.07.00 programs were used to perform post-run analyses. A ^{13}C-short chain fatty acids stool mixture (Merck Life Science, Poole, UK) was used as the internal standard to normalise all spectra obtained prior to analyses.

References

1. Frank L, Kleinman L, Farup C, Taylor L, Miner PJ. 1999. Psychometric validation of a constipation symptom assessment questionnaire. Scand J Gastroenterol 34:870–877.

2. Jaudszus A, Zeman E, Jans T, Pfeifer E, Tabori H, Arnold C, Michl RK, Lorenz M, Beiersdorf N, Mainz JG. 2019. Validity and Reliability of a Novel Multimodal Questionnaire for the Assessment of Abdominal Symptoms in People with Cystic Fibrosis (CFAbd-Score). Patient 12:419–428.

3. Marsh R, Gavillet H, Hanson L, Ng C, Mitchell-Whyte M, Major G, Smyth AR, Rivett D, van der Gast C. 2022. Intestinal function and transit associate with gut microbiota dysbiosis in cystic fibrosis. J Cyst Fibros 21:506–513.

4. Walters W, Hyde ER, Berg-lyons D, Ackermann G. 2016. Improved Bacterial 16S rRNA Gene (V4 and V4-5) and Fungal Internal Transcribed Spacer Marker Gene Primers for Microbial Community Surveys. mSystems 1:e00009-15.

5. Parks DH, Chuvochina M, Waite DW, Rinke C, Skarshewski A, Chaumeil PA, Hugenholtz P. 2018. A standardized bacterial taxonomy based on genome phylogeny substantially revises the tree of life. Nat Biotechnol 36:996.

Supplementary Results

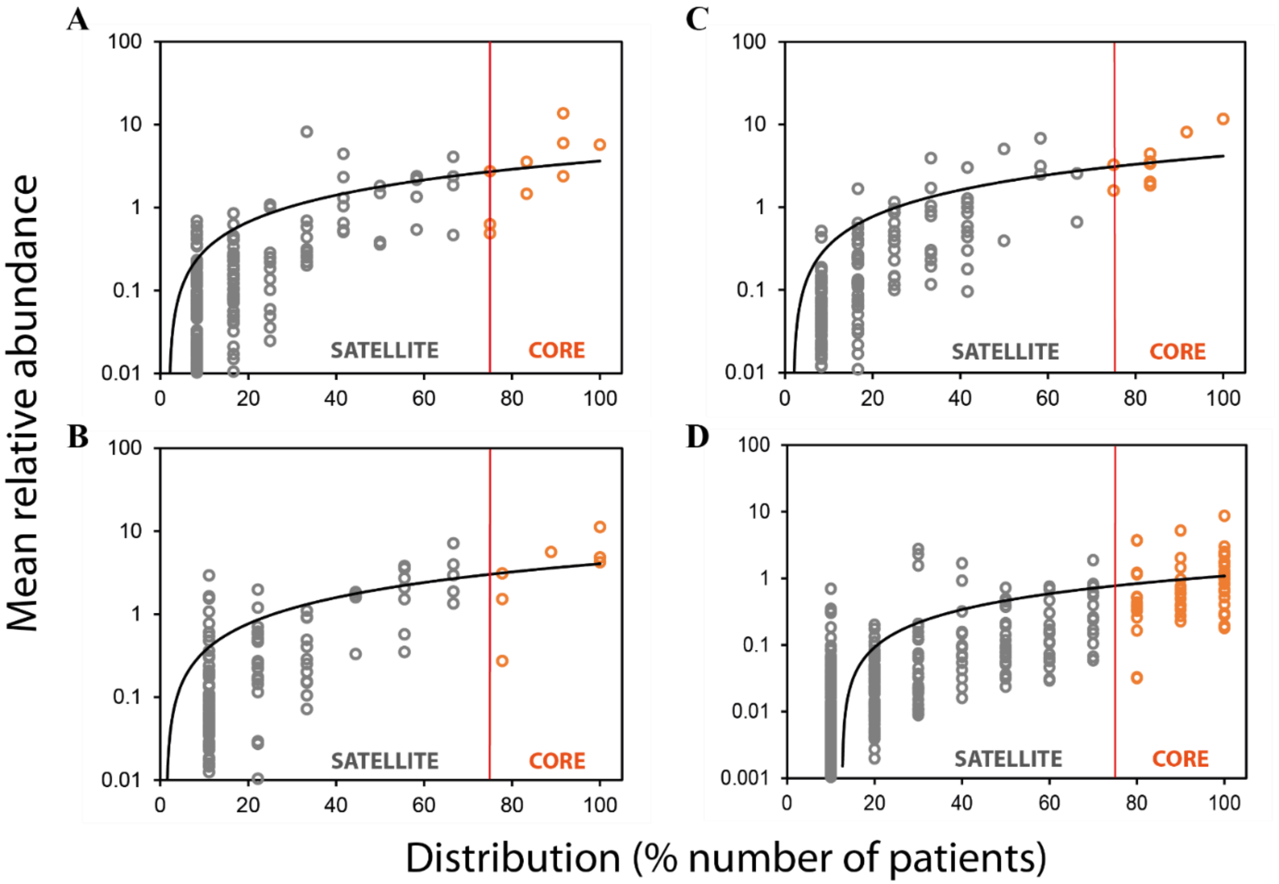

Figure S1. Distribution and abundance of bacterial taxa across treatment stages (A-C) and healthy control participants (D). Given is the percentage number of patients harbouring each bacterial taxon, plotted against the mean relative abundance across those samples. Core taxa are depicted by the orange circles and fall in the upper quartile of distribution, separated by the red vertical line at 75% distribution. Satellite taxa (grey) are all samples below this distribution. Distribution-abundance relationship regression statistics: (a) r^2 = 0.45, $F_{1,196}$ = 152.6 , P < 0.0001. (b) r^2 = 0.60, $F_{1,175}$ = 245.9, P < 0.0001. (c) r^2 = 0.57, $F_{1,175}$ = 245.9, P < 0.0001. (d) r^2 = 0.28, $F_{1,465}$ = 182.8 , P < 0.0001.

Table S1. Core taxa between different treatment phases.

Taxa	Baseline Prev.	Baseline Rel. abundance	Placebo Prev.	Placebo Rel. abundance	Tez/Iva Prev.	Tez/Iva Rel. abundance	Controls Prev.	Controls Rel. abundance
Blautia wexlerae	91.67	13.67	100.00	11.23	100.00	11.66	100.00	2.99
Escherichia coli	58.33	2.39	66.67	3.97	75.00	1.59	90.00	2.02
Blautia faecis	66.67	2.37	77.78	3.09	83.33	3.36	100.00	1.53
Dorea longicatena	83.33	3.55	100.00	4.22	83.33	4.44	100.00	0.97
Fusicatenibacter saccharivorans	100.00	5.70	88.89	5.60	83.33	3.54	100.00	2.36
Enterococcus 24	75.00	2.74	66.67	1.34	58.33	2.50	10.00	0.30
Streptococcus 27	91.67	2.38	66.67	1.87	83.33	2.02	100.00	0.19
Anaerobutyricum hallii	91.67	5.97	100.00	4.82	91.67	8.09	100.00	1.77
Anaerostipes hadrus	66.67	4.06	55.56	3.79	75.00	3.26	100.00	1.27
Parabacteroides distasonis	83.33	1.46	77.78	1.52	83.33	1.84	90.00	0.97
Erysipelatoclostridium ramosum	75.00	0.63	77.78	0.27	41.67	0.50	20.00	0.04
Agathobaculum 309	75.00	0.49	44.44	0.33	41.67	0.18	90.00	0.50

Given is prevalence, the percent number of samples a given core taxon was detected in, and average relative abundance across those samples. Taxon names are derived from condensed ASVs of the same species. ASV numbers have been used to differentiate between taxa within the same genus that could not be identified at the species level. Core taxa are highlighted orange, whereas satellite taxa are grey. Given the length of the ribosomal sequences analysed, species identities should be considered putative.

Table S2. Kruskall-Wallis tests of bacterial alpha diversity utilising Fishers alpha index.

	Microbiota		Core taxa		Satellite taxa	
Baseline vs Placebo	K (Observed value)	1.823	K (Observed value)	7.293	K (Observed value)	1.136
	K (Critical value)	3.841	K (Critical value)	3.841	K (Critical value)	3.841
	DF	1	DF	1	DF	1
	p-value (one-tailed)	0.177	p-value (one-tailed)	0.007	p-value (one-tailed)	0.286
Baseline vs Tez/Iva	K (Observed value)	0.053	K (Observed value)	0.083	K (Observed value)	0.213
	K (Critical value)	3.841	K (Critical value)	3.841	K (Critical value)	3.841
	DF	1	DF	1	DF	1
	p-value (one-tailed)	0.817	p-value (one-tailed)	0.773	p-value (one-tailed)	0.644
Placebo vs Tez/Iva	K (Observed value)	0.247	K (Observed value)	4.247	K (Observed value)	0.081
	K (Critical value)	3.841	K (Critical value)	3.841	K (Critical value)	3.841
	DF	1	DF	1	DF	1
	p-value (one-tailed)	0.619	p-value (one-tailed)	0.039	p-value (one-tailed)	0.776

Table S3. Bacterial ANOSIM summary statistics between treatment phases, utilising the Bray-Curtis index.

	**Microbiota**		**Core taxa**		**Satellite taxa**	
Baseline	R value:	-0.0652	R value:	0.0768	R value:	-0.0392
vs	*p* same:	0.8216	*p* same:	0.1373	*p* same:	0.6724
Placebo	Bonferroni-corrected p value	0.8366	Bonferroni-corrected p value	0.143	Bonferroni-corrected p value	0.6674
	permutation *N*:	9999	permutation *N*:	9999	permutation *N*:	9999
Baseline	R value:	-0.0720	R value:	0.0359	R value:	0.0484
vs	*p* same:	0.9328	*p* same:	0.1987	*p* same:	0.1946
Tez/Iva	Bonferroni-corrected p value	0.9363	Bonferroni-corrected p value	0.2036	Bonferroni-corrected p value	0.2006
	permutation *N*:	9999	permutation *N*:	9999	permutation *N*:	9999
Placebo	R value:	-0.0323	R value:	0.0686	R value:	0.0047
vs	*p* same:	0.6349	*p* same:	0.1529	*p* same:	0.4308
Tez/Iva	Bonferroni-corrected p value	0.6423	Bonferroni-corrected p value	0.1548	Bonferroni-corrected p value	0.437
	permutation *N*:	9999	permutation *N*:	9999	permutation *N*:	9999

Table S4. Kruskal-Wallis tests of bacterial alpha diversity utilising Fishers alpha index

	Control vs Baseline		Control vs Placebo		Control vs Tez/Iva	
Micro	K (Observed value)	15.652	K (Observed value)	13.500	K (Observed value)	15.652
	K (Critical value)	3.841	K (Critical value)	3.841	K (Critical value)	3.841
	DF	1	DF	1	DF	1
	p-value (one-tailed)	<0.0001	p-value (one-tailed)	0.0002	p-value (one-tailed)	<0.0001
Core	K (Observed value)	15.652	K (Observed value)	13.500	K (Observed value)	15.652
	K (Critical value)	3.841	K (Critical value)	3.841	K (Critical value)	3.841
	DF	1	DF	1	DF	1
	p-value (one-tailed)	<0.0001	p-value (one-tailed)	0.0002	p-value (one-tailed)	<0.0001
Sat	K (Observed value)	15.652	K (Observed value)	13.500	K (Observed value)	15.652
	K (Critical value)	3.841	K (Critical value)	3.841	K (Critical value)	3.841
	DF	1	DF	1	DF	1
	p-value (one-tailed)	<0.0001	p-value (one-tailed)	0.0002	p-value (one-tailed)	<0.0001

Table S5. Bacterial ANOSIM summary statistics between treatment stages and controls, utilising the Bray-Curtis index.

	Healthy vs Baseline		Healthy vs Placebo		Healthy vs Tez/Iva	
Micro	R value:	0.5914	R value:	0.5646	R value:	0.6308
	p same:	0.0001	*p* same:	0.0001	*p* same:	0.0001
	Bonferroni-corrected p value	0.0001	Bonferroni-corrected p value	0.0002	Bonferroni-corrected p value	0.0001
	permutation *N*:	9999	permutation *N*:	9999	permutation *N*:	9999
	**Healthy vs Baseline**		**Healthy vs Placebo**		**Healthy vs Tez/Iva**	
Core	R value:	0.568	R value:	0.4809	R value:	0.7156
	p same:	0.0001	*p* same:	0.0001	*p* same:	0.0001
	Bonferroni-corrected p value	0.0001	Bonferroni-corrected p value	0.0001	Bonferroni-corrected p value	0.0001
	permutation *N*:	9999	permutation *N*:	9999	permutation *N*:	9999
	**Healthy vs Baseline**		**Healthy vs Placebo**		**Healthy vs Tez/Iva**	
Sat	R value:	0.7736	R value:	0.89	R value:	0.7634
	p same:	0.0001	*p* same:	0.0001	*p* same:	0.0001
	Bonferroni-corrected p value	0.0001	Bonferroni-corrected p value	0.0001	Bonferroni-corrected p value	0.0001
	permutation *N*:	9999	permutation *N*:	9999	permutation *N*:	9999

Table S6. Analytical parameters for SCFA analysis with GC-MS

SCFA	t_R (min)	Target ion (m/z)	Confirmation ion (m/z)
**Acetic acid**	2.52	117	75
**Propionic acid**	3.81	131	75
**Iso-butyric acid**	4.39	145	117
**Butyric acid**	5.22	145	117
**Iso-valeric acid**	6.07	159	117
**Valeric acid**	6.85	159	117
**4-methylvaleric acid**	7.86	173	117
**Hexanoic acid**	8.41	173	117
**Heptanoic acid**	9.9	187	117
**^{13}C-acetic acid**	2.52	119	77
**^{13}C-propionic acid**	3.81	134	77
**^{13}C-butyric acid**	5.22	149	119

t_R - Retention time

Table S7. Short chain fatty acid compositional ANOSIM summary statistics between treatment stages and controls, utilising the Bray-Curtis index.

**Healthy vs Baseline**		**Healthy vs Placebo**		**Healthy vs Tez/Iva**	
R value:	0.2518	R value:	0.242	R value:	0.108
p same:	0.0034	*p* same:	0.0047	*p* same:	0.041
Bonferroni-corrected p value	0.0050	Bonferroni-corrected p value	0.0051	Bonferroni-corrected p value	0.0459
permutation *N*:	9999	permutation *N*:	9999	permutation *N*:	9999
**Baseline vs Placebo**		**Baseline vs Tez/Iva**		**Placebo vs Tez/Iva**	
R value:	-0.0423	R value:	0.0360	R value:	-0.0208
p same:	0.6966	*p* same:	0.1911	*p* same:	0.5437
Bonferroni-corrected p value	0.7043	Bonferroni-corrected p value	0.1933	Bonferroni-corrected p value	0.5464
permutation *N*:	9999	permutation *N*:	9999	permutation *N*:	9999

Table S8. SIMPER results between SCFA relative levels between controls and pwCF.

Controls vs Baseline

SCFA	Av. dissim	Contrib. %	Cumulative %	Mean Healthy	Mean baseline
Butyric	9.935	34.6	34.6	30.9	41.6
Acetic	6.991	24.35	58.95	32.7	34.2
Propionic	4.899	17.06	76.01	20.7	17.8
Valeric	2.388	8.317	84.33	5.41	0.634
Isovaleric	1.591	5.54	89.87	3.7	3.07
Isobutyric	1.357	4.727	94.59	3.5	2.13
Hexanoic	1.239	4.314	98.91	2.6	0.426
Heptanoic	0.2202	0.7667	99.67	0.443	0.00833
4-methylvaleric	0.09348	0.3256	100	0.091	0.148
				**Dissimilarity**	**28.71**

Controls vs Placebo

SCFA	Av. dissim	Contrib. %	Cumulative %	Mean Healthy	Mean placebo
Butyric	7.031	32.66	32.66	30.9	43.1
Propionic	4.41	20.49	53.14	20.7	23
Acetic	4.315	20.04	73.19	32.7	28.1
Valeric	1.993	9.259	82.45	5.41	1.69
Hexanoic	1.25	5.805	88.25	2.6	0.176
Isobutyric	1.132	5.257	93.51	3.5	1.56
Isovaleric	1.115	5.181	98.69	3.7	2.4
Heptanoic	0.2197	1.02	99.71	0.443	0.0111
4-methylvaleric	0.06227	0.2892	100	0.091	0.0944
				**Dissimilarity**	**21.53**

Controls vs Tez/Iva

SCFA	Av. dissim	Contrib. %	Cumulative %	Mean Healthy	Mean Tez/Iva
Butyric	7.08	28.44	28.44	30.9	31.4
Acetic	6.069	24.38	52.83	32.7	34.6
Propionic	5.101	20.5	73.32	20.7	24.1
Valeric	2.048	8.228	81.55	5.41	1.91
Isovaleric	1.738	6.983	88.53	3.7	4.61
Isobutyric	1.329	5.34	93.87	3.5	3.2
Hexanoic	1.25	5.021	98.89	2.6	0.172
Heptanoic	0.2197	0.8827	99.77	0.443	0.0117
4-methylvaleric	0.05608	0.2253	100	0.091	0.0842
				**Dissimilarity**	**24.89**

Table S9. Differences in relative levels of SCFAs between healthy control subjects and pwCF during different treatment phases.

	P value		
**SCFA**	**Baseline**	**Placebo**	**Tez/Iva**
Acetic	0.742	0.253	0.210
Propionic	0.166	0.327	0.262
Iso-butyric	0.048	0.009	0.429
Butyric	0.210	0.022	0.843
Iso-valeric	0.210	0.165	0.895
Valeric	0.000	0.003	0.006
4-methylvaleric	0.086	0.253	0.323
Hexanoic	0.099	0.060	0.013
Heptanoic	0.040	0.060	0.086

Given are *p* values obtained following Kruskal-Wallis testing of the relative levels of SCFAs within the healthy control samples and all treatment phases. Dark green - $p < 0.001$, Green - $p < 0.01$, Light Green - $p < 0.05$, Yellow – $p < 0.1$.

Table S10. Summary statistics for PAC-SYM scores across Tezacaftor/Ivacaftor and off-treamtent samples.

Variable	Observations	Minimum	Maximum	Mean	Std. deviation
Tezacaftor/Ivacaftor	12	0.000	1.000	0.409	0.385
Off-treatment	14	0.000	2.330	0.636	0.625

Kruskal-Wallis test / Two-tailed test:

K (Observed value)	0.730
K (Critical value)	3.841
DF	1
p-value (one-tailed)	0.393
alpha	0.050

An approximation has been used to compute the *P*-value.

Table S11. Summary statistics for CFAbd scores across Tezacaftor/Ivacaftor and off-treamtent samples.

Variable	Observations	Minimum	Maximum	Mean	Std. deviation
Off-treatment	14	1.400	43.100	20.829	12.583
Tezacaftor/Ivacaftor	12	1.400	45.100	15.483	12.910

t-test for two independent samples / Two-tailed test:

Difference	5.345
t (Observed value)	1.067
|t| (Critical value)	2.064
DF	24
p-value (Two-tailed)	0.297
alpha	0.050
